# Rehabilitation of an Auricular Defect Using Surgical Stent

**Published:** 2018-01

**Authors:** Mahnaz Arshad, Gholamreza Shirani, Sina Refoua

**Affiliations:** 1 Prosthodontics Department, Dental Implant Center, School of Dentistry, Tehran University of Medical Sciences, Tehran, Iran;; 2 Department of Oral and Maxillofacial Surgery, School of Dentistry, Tehran University of Medical Sciences, Tehran, Iran;; 3 School of Dentistry, Tehran University of Medical Sciences, Tehran, Iran

**Keywords:** Implant, Dental prosthesis, Ear implant, Ossicular replacement

## Abstract

Reconstruction of a facial defect is a complex modality either surgically or prosthetically, depending on the site, size, etiology, severity, age, and the patient’s expectation. The loss of an auricle, in the presence of an auditory canal, affects hearing, because the auricle gathers sound and directs it into the canal. The auricle acts as a resonator to slightly amplify lower frequency sounds and helps to localize sounds, especially in conjunction with the other ear. Osseointegrated implants have an important role in the prosthetic reconstruction of patients with craniofacial defects. The main indications of this treatment plan are lack of local tissue for autogenous reconstruction, previous reconstruction failure and selection of this technique by the patient. This paper presents a clinical case and advantages of the osseointegrated implant technique for retention of auricular prostheses.

## INTRODUCTION

Quality of life can be affected easily by physical defects, especially with maxillofacial defects. Different factors lead to auricular defects including traumatic accidents or congenital defects represented as malformation of the organ.^1^ Congenital auricular defects in children are often related to Treacher Collins Syndrome (TCS) or Hemifacial Microsomia (HFM), which is the second most common craniofacial malformation after cleft lip and palate.^2^^,^^3^ Various types of external ear malformation can be found in such patients from aplasia in complete form to displaced and distorted pinna.^4^

The complex shape and size of human ear makes the surgical reconstruction of auricular defects one of the main challenging issues for the surgeon. Because of the special structure of the ear, regarding its complicated shape and its dominant location, its esthetic reconstruction is a big deal. Due to this issue for a surgeon is necessary to have a perfect insight into ear’s external anatomy and cephalometric parameters. The helix and antihelixe, central conchal complex, and inferiorly placed lobule, organized auricular cartilage structure.^5^^,^^6^

The implant retained prosthesis of ear in comparison with other conventional treatments such as using of adhesive material, prosthesis installed on spectacles or through the use of undercuts is more reliable and durable.^7^ The aim of this article is to introduce a presurgically diagnostic procedure to specify the implants placement location in small children who are in need with auricular prosthesis. 

## CASE REPORT

**Fig. 1:**
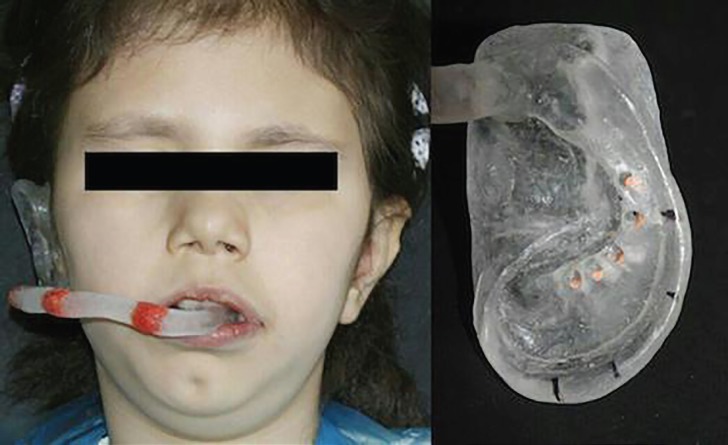
**A. **12-year-old female patient with right ear loss. Initial clinical presentation. **B.** anterior view.

A 12-year-old girl presented with congenitally lost in right ear and strabismus was referred to the Department of Prosthodontics at Tehran University of Medical Sciences (Figure 1). The patient hearing condition was evaluated and no disorder was protected. To fabricate an auricular prosthesis we followed some particular steps including: (i) duplicating the defective area and replicating the intact side; (ii) creating a clay or wax sculpture of the future prosthesis; (iii) producing a 3-piece radiographic and surgical stent with clear acrylic resin; (iv) using of radio-opaque landmarks to determine implants location, (v) taking CT scanning, (vi) analysis the radiograph and bone, and (vii) surgery, fabricating and hand painting the final prosthesis. Informed consent was taken from parents of the patient participated in this study. A written consent from the patient’s legal guardian was obtained in order to publish her images too. 

**Fig. 2:**
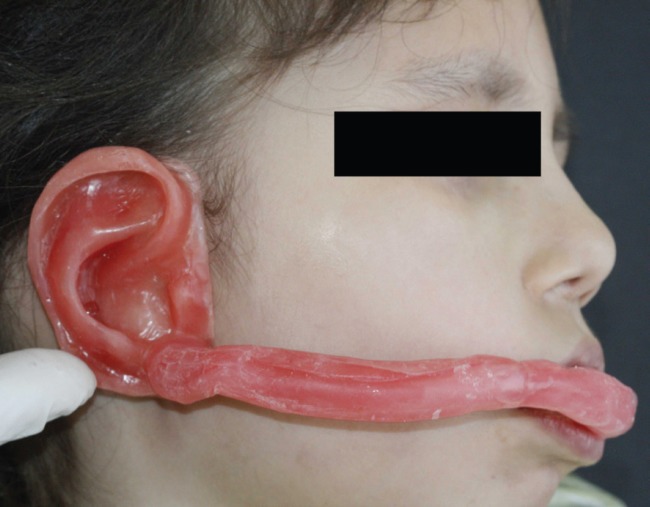
Waxed up ear trying in.

**Fig. 3:**
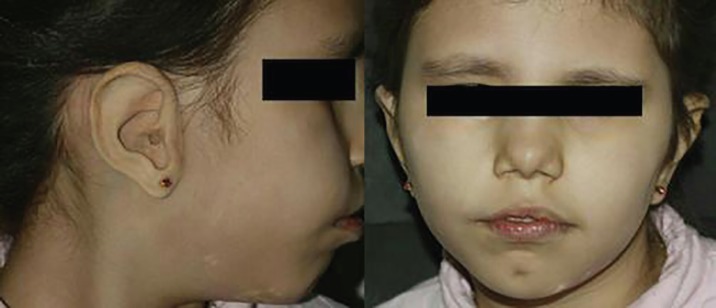
Surgical stent made from clear acrylic resin.

Primary impression from left and residual right ears and jaws were done. Upon left ear, right ear was waxed up and tried in (Figure 2). To obtain an index for implants location, two holes prepared in the wax sculpture in eleven and seven o’clock regions,^8^ and gutta (Diadent. Seoul, South Korea) was put in the holes. With the stent in place, CT scan was taken and bone and site of implantation was investigated on CT images and surgical stent was fabricated with clear acrylic resin (Beredent GmbH and co. KG. Germany) (Figure 3).

**Fig. 4:**
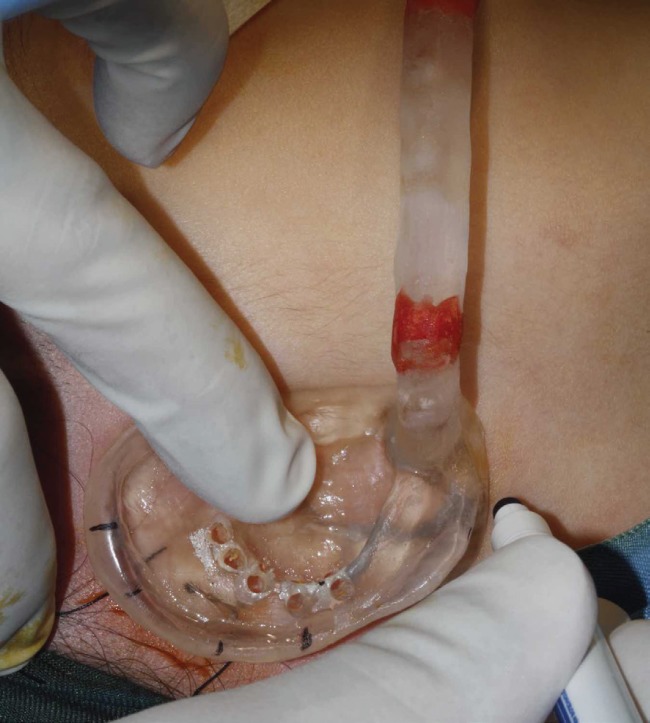
Surgical stent is used to determine implants location and angulation.

An incision was made posterior to external acoustic meatus. Its direction was determined through the holes which were prepared on the splint before surgery, and the temporal bone was exposed. In order to prevent osteocytes get damaged and providing direct contact between implant and bone (osseointegration), the surgeon must apply a gentle technique.^9^ To achieve the most desired implants angulations and placements and adequate spacing, the surgeon used surgical template. Furthermore, to assure the sufficiency of bone volume for implant placement, bone quantity was evaluated at the time of surgery. Under general anesthesia, two implants; 3.3/5.5 mm (ITI, Straumann AG, Basel, Switzerland) were inserted in seven and eleven o’clock holes (Figure 4).

**Fig. 5:**
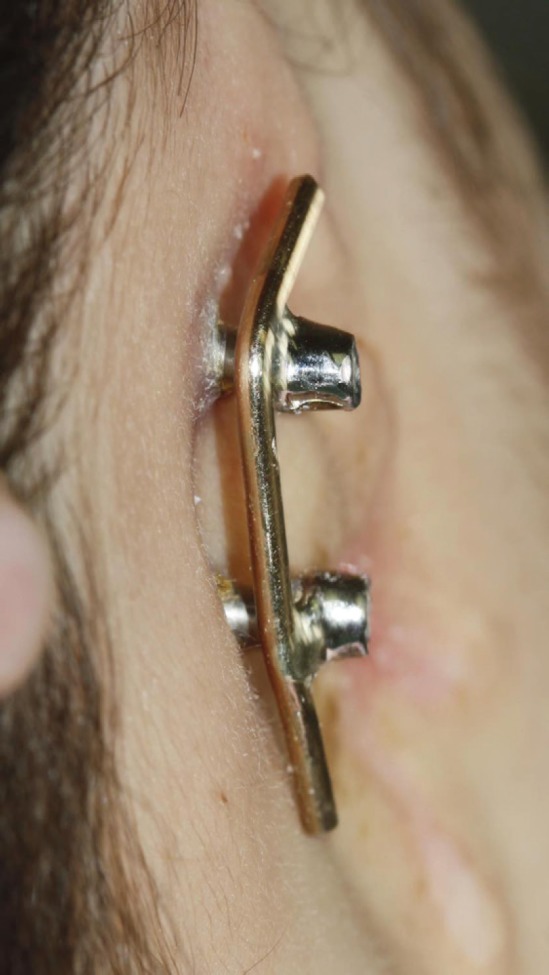
Bar retention clip system.

To further improve aesthetically the final ear prosthesis, residual right ear was resected. According to better coetaneous adaptation that fixture mount has preferred to use fixture mount instead of healing. Also in this method, there was no need for second surgery. Ten days later, the patient was followed and sutures were removed. After healing period, abutments (ITI, Straumann AG, Basel, Switzerland) were put in place and final impression with additional polyvinyl siloxane (Panasil, kettenbach GmbH & co. KG) by close tray method at abutment level was done. After waxing up the bar, it was removed gently from the analogues and invested. It was then cast in metal (Deoband alloy, Aurident. inc.USA). The cast bar was finished and polished. The bar was now tried onto the implants placed into the patient’s skull (Figure 5). 

**Fig. 6:**
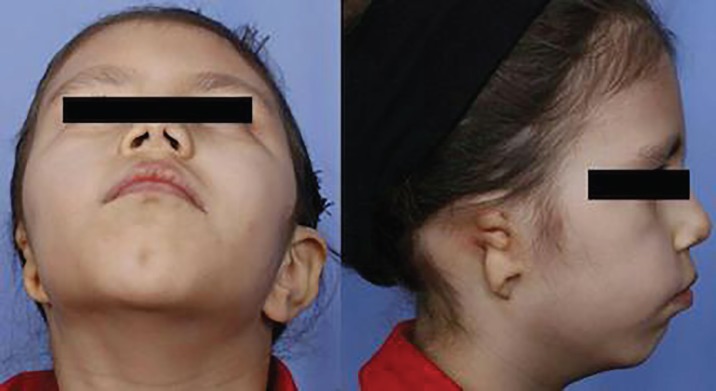
**A **and **B:** Patient after reconstruction.

By using dental wax or modeling clay or both of them, the prosthetic ear was sculpted on the model. To make sure of the right dimension of the sculpted ear and its compatibility with patient facial features, the sculpted ear was tried on the patient. The mold was packed with silicone rubber, which is the material of choice for most maxillofacial prosthesis at the moment. After polymerization of the silicone, the prosthesis was finished with abrasive wheels. Extrinsic coloring was added to the prosthesis with the patient present so that the color, hue and tone of the patient’s skin could be matched perfectly. The prosthesis was then placed on the patient (Figure 6). The prosthesis could be used constantly and there was no need for rinsing. 

## DISCUSSION

The benefits of early treatment, including reconstruction of facial deformities, to the psychological wellbeing for children when they reach school age has been demonstrated.^8^^,^^10^

The implant supported prosthesis works very well. After the patient’s body accepted the implants, the entire team was confident that the whole treatment plan would be a resounding success. With using the healings instead of fixture mounts, patients’ costs were lowered and there was no need for patient to undergo second surgery. All the tissue tags were removed so obtained aesthetic was preferable, and prosthesis had more compatibility with skin, and it was more symmetrical. The most difficult task of the whole treatment was to match the color of the prosthesis exactly to that of the patient’s skin. With experience the task would however become much easier.

In comparison with adhesive-retained prosthesis, in this method, prosthesis needs less home care maintenance. Adhesive-retained prosthesis needs more patience and precision of the wearer to obtain better appearance. Silicon-based adhesives should be cleaned which may increases the chance of prosthesis marginal distortion, and allergic contact dermatitis in intended area.^2,11 ^Wearing and tearing of retentive components of implant-retained prosthesis is the major complication.^10 ^Blood flow in temporal bone is strongly related with the patient age so the age factor plays a critical role in treatment success.^5 ^Upon this procedure, we underlined the exact presurgically analysis and surgical stent that could achieve to the best retention and aesthetic outcome.
